# A Questionnaire Study on the Attitude towards Death of the Nursing Interns in Eight Teaching Hospitals in Jiangsu, China

**DOI:** 10.1155/2019/3107692

**Published:** 2019-09-16

**Authors:** Fengqin Xu, Kun Huang, Yinhe Wang, Yuzi Xu, Liang Ma, Yang Cao

**Affiliations:** ^1^The First Affiliated Hospital of Kangda College of Nanjing Medical University, The First People's Hospital of Lianyungang, The Affiliated Lianyungang Hospital of Xuzhou Medical University, Lianyungang 222000, Jiangsu, China; ^2^Kangda College of Nanjing Medical University, Lianyungang 222000, Jiangsu, China; ^3^Department of Orthopaedic Surgery, Nanjing Drum Tower Hospital, The Affiliated Hospital of Nanjing University Medical School, Nanjing 210008, Jiangsu, China; ^4^Department of Stomatology, Zhejiang University School of Medicine, Hangzhou 310058, Zhejiang, China; ^5^Clinical Epidemiology and Biostatistics, School of Medical Sciences, Örebro University, Örebro 70182, Sweden

## Abstract

**Introduction:**

Nurses play an important role in caring for dying patients. However, little is known about the attitude towards death of the registered nurses in China.

**Materials and Methods:**

A knowledge, attitude, and the practice (KAP) survey using standardized questionnaires was conducted at eight teaching hospitals in Jiangsu Province, China. In total, 366 nursing interns were recruited and 357 turned in valid response. Data about the interns' demographic characteristics and their attitudes to death in five domains, including fear of death, death avoidance, natural acceptance, approach acceptance, and escape acceptance, were collected.

**Results:**

Compared to the norms, the nursing interns had statistically significantly higher scores in the domains death avoidance, approach acceptance, and fear of death (14.9 vs. 11.1, 26.2 vs. 24.2, and 20.3 vs. 19.0, respectively); however, statistically significantly lower scores were in the domains natural acceptance and escape acceptance (18.4 vs. 22.0, and 13.6 vs. 15.1, respectively). Religious belief, experience of a deceased relative in family, death education, and family atmosphere of discussing death are positively associated with one or more domains of attitude towards death.

**Conclusion:**

The positive attitude towards death and death education before clinical practice are helpful for nursing interns when they care for dying patients. In general, the scores of attitude towards death are at a moderate level in the surveyed Chinese nursing interns. The death education for nursing students needs to be reinforced in China.

## 1. Introduction

Failure to discuss death as part of normal life may have a number of consequences, including fear of the process of dying, a lack of awareness and openness between close family members, and lack of knowledge about how to request and provide services when a person is dying. It is necessary to better understand death, accept, and prepare for it with a mature attitude [1, 2].

Those nearing the end of their lives deserve to be given optimum care, attention, compassion, and consideration [3]. Attitude towards death is one of the most important factors that can influence the behavior of health professionals. Undoubtedly, nurses play an important role in caring for the dying patients. However, there are consistent reports indicating that new nurses have stronger fear of death and more negative attitude towards end-of-life patient care than experienced nurses [4].

Attitudes towards death are, to some extent, based upon people's faith, ethnicity, education, and other socioeconomic and religious characteristics [5]. Nursing students and nurses in different countries may have different attitudes towards death or caring dying patients. In eastern countries like Japan, where people in general believe in the existence of the life after death [6], the registered nurses have important roles in providing end-of-life care to older adults [7]. In Iran, nurses towards Islam believe in resurrection after earthly life; therefore, most of them stated that one of the reasons of relaxation when facing the death was their belief to the life after the death [8]. These studies demonstrated that nurses' attitude towards death was partially explained by the beliefs in religion. In western countries, thoughts and attitudes towards death have been under the influence of their Greek/Latin roots and then by Christianity, Enlightenment, and Modernity. In Israel, a study on oncology nurses'personal attitude towards death implies that culture and religion might play important roles in the development of the attitude towards the care of dying patients [9]. Although both civilizations moved through broadly similar stages, some influential contextual factors have been very influential in shaping the difference in attitude towards death between them [10]. For example, the weakest factor of the four factors associated with the fear of another person's dying in a Hong Kong sample was the strongest one in a German sample [11]. 

Work experience and continuing education also play roles in shaping nurses' attitude towards death. More exposure and experience in working with dying patients correlated to more positive attitude in caring for dying patients. The experienced nurses appeared to have more flexible attitude, and adhered less strictly to the palliative treatment than the inexperienced students did. A study conducted in New York indicated that older nurses of a Comprehensive Cancer Center had a more positive attitude towards caring for dying patients than their younger counterparts. In contrast, nurses who lacked such experience showed more negative attitude, and reported more feelings of fear for death [12]. The nurses who attended the seminars on end-of-life care were more likely to have better attitude towards caring for dying patients than those who did not attend [7]. Compared to the male counterparts, the female nursing students showed more positive attitude towards caring for dying patients during treatment and after death [13]. Nurses from different departments also show different attitudes towards death and different contacts with terminally ill patients. For example, in Spain, the nephrology nurses showed positive attitude towards care of dying renal patient, and the attitude was associated with their experience in caring for dying patients, previous training in end-of-life care, and willingness to discuss the end-of-life care issues [14].

Because of the traditional beliefs, to Chinese, it is taboo and discouraged to discuss death in daily life. In general, Chinese people tend to use metaphorical expressions and/or rhetorical pictures to convey meanings and emotions implicitly, especially when they talk about death. The knowledge of these death metaphors and pictures can provide a better understanding of Chinese personal perceptions of death [15]. However, up to know, little is known about the attitude towards death of the registered nurses in China [16], let alone the nursing interns who have little experience in caring for dying patients. Nursing studentsand nurses' attitude towards caring for the dying patients need to be investigated in China with an aging population.

In Kangda College of Nanjing Medical University, the students of the four-year program of Bachelor of Science in Nursing begin their internship in the fourth year of their study. The death education is given throughout the first three years both in class and hospital exercitation before internship, by using lectures/courses, focus group discussion, and/or problem based learning. The contents of the education include Death and Dying, Evolution and Development of Humans' Knowledge of Death, Progress of Life Cycle, Meaning of Death, Death Epidemiology, Socioeconomic Issues of Death, and Hospice and Palliative Care [17]. The present study aimed to investigate and assess the attitude of nursing interns caring for dying patients in eight teaching hospitals of Kangda College of Nanjing Medical University in Jiangsu Province, China.

## 2. Materials and Methods

### 2.1. Setting

This study was conducted between July, 2017 and September, 2018 in eight teaching hospitals in Jiangsu Province, China. The approval for conducting this study was obtained from the Institutional Ethics Committee of the First People's Hospital of Lianyungang (approval number: KY20170701001).

### 2.2. Study Design

The study is a questionnaire survey on the knowledge, attitude, and practice (KAP) using standardized questionnaires. The convenience sampling method was used to recruit 366 nursing interns from the eight teaching hospitals affiliated to Kangda College of Nanjing Medical University in Jiangsu Province, China. The nursing interns had worked more than 5 months in the teaching hospitals, and participated in the survey voluntarily with signed consent form. As a descriptive and explorative study, no predefined power and type I error were used for sample size determination.

### 2.3. Data Collection

Two standardized questionnaires were used in the KAP survey. One collected the data regarding the nursing interns' general information, including gender, age, religious belief, the only child in the family, living with single parent, living area, education level, perception of mind and body, experience in caring for dying patients, experience of a deceased relative in family, death education, and family atmosphere of discussing death. The other questionnaire was the Death Attitude Profile-revised (DAP-R) [18]. The reliability and validity of the Chinese version DAP-R had been validated in previously studies [19, 20]. DAP-R reliability values (Cronbach's *α*) were 0.84, 0.87, 0.63, 0.89, and 0.9 for the five domains of attitude towards death, i.e. fear of death, death avoidance, natural acceptance, approach acceptance, and escape acceptance, respectively [18]. Fear of death is negative thoughts and emotions such as fear and scare when people faced death. Death avoidance is that people do not want to think and discuss the things related to death. Neutral acceptance is that people do not fear but also do not welcome death. They just regard it as a natural process of life. Approach acceptance is that people regard death as a way to happiness. They believe that there is a happy life after death. Escape acceptance is that people regard death as a pain relief in life.

The Chinese version of DAP-R included 32 items of the above five domains of attitude towards death. Each item is scored using five-point Likert scale from strongly disagree (1 point) to strongly agree (5 points). Therefore, the total scores of questionnaire may range from 32 to 160 points, with a higher score indicating a more positive attitude towards caring for dying patients.

Previous study showed that the Cronbach's coefficients of most domains in the Chinese DAP-R were above 0.7, and the split-half correlation coefficient was 0.864, which indicated that the Chinese DAP-R had good internal consistency. The overall reliability of the questionnaire was 87.5% [19].

The researchers gave detailed introduction and guidance to the nursing interns on how to fill in the questionnaires before the survey. Questionnaires were collected immediately after the nursing interns completed them. Of the 366 recruited nursing interns, 357 valid questionnaires were returned, and the response rate was 97.5%.

### 2.4. Statistical Analysis

The attitude scores were summarized using mean and standard deviation (SD). The difference between the groups was tested using the Student's *t*-test for two groups, and one-way analysis of variance (ANOVA) for multiple groups, or nonparametric test for the scores that were not normally distributed. A two-sided *p*-value <0.05 is considered statistically significant. The generalized linear regression model was used to evaluate the impact of the potential influential factors on the attitude scores. The backward stepwise method was used for variable selection, with a *p*-value of 0.05 for entering and a *p*-value of 0.10 for removal. The adjusted regression analyses were also conducted by including the norms [21] in the models of the Chinese undergraduate nursing student. All the analyses were conducted in the statistical software SPSS version 25 (IBM, Armonk, New York).

## 3. Results

### 3.1. The Status quo of the Nursing Interns' Attitude towards Death

The rank of the five domains' average scores of the nursing interns' attitude towards death from high to low is: natural acceptance, death avoidance, fear of death, escape acceptance and approach acceptance (Table 1). Compared to the norms of the Chinese undergraduate nursing students, the nursing interns in our study had statistically significantly higher scores in the domains death avoidance, approach acceptance, and fear of death (14.9 vs. 11.1, 26.2 vs 24.2, and 20.3 vs. 19.0, respectively); however, statistically significantly lower scores in the domains natural acceptance and escape acceptance (18.4 vs. 22.0, and 13.6 vs. 15.1, respectively) (Table 1).

### 3.2. Subgroup Analysis by Demographic Characteristics

As shown in Table 2, in general, no statistically significant difference was found between sexes and age groups. However, male nursing interns had a higher average score in the domain escape acceptance (15.5 vs. 13.5), and the youngest nursing interns had the lowest average score (17.9) in the domain natural acceptance. The nursing interns having religious belief showed lower scores in all the domains and statistically significant in the domains fear of death (20.1 vs. 22.4), approach acceptance (25.7 vs. 31.0), and escape acceptance (13.4 vs. 16.0). Although the interns being the only child in the family had statistically significantly lower scores in the domain natural acceptance (18.2 vs. 18.8), we do not think the difference clinically significant. No statistically significant difference was found in the attitude towards death between the interns whether living with single parent or not, between the three living areas, and between the education levels. The interns having poor perception of mind and body had the highest average score (19.0) in the domain natural acceptance. Comparing with the interns having no experience in caring for dying patients or of a deceased relative in family, the experienced ones had statistically significant higher scores in the domain natural acceptance (18.6 vs. 17.8, and 18.7 vs. 17.8, respectively). The interns having death education showed higher scores in the domain natural acceptance (19.0 vs. 17.8), but lower scores in the domains fear of death (19.5 vs. 21.1), death avoidance (14.2 vs. 15.6), and escape acceptance (13.2 vs. 14.0). The interns from the family having open and free atmosphere of discussing death had the highest average score in domain natural acceptance (19.8) but the lowest average score in the domain death avoidance (13.8).

### 3.3. Influential Factors of the Nursing Interns' Attitude Towards Death

In the generalized linear regression models, scores of the five attitude domains were treated as the dependent variables, and gender, age, religious beliefs, and other demographic characteristics variables were included as explanatory variables. Both adjusted and non-adjusted results (Table 3) indicate that religious belief and death education are positively and negatively associated with fear of death, respectively. Both death education and family atmosphere of discussing death are negatively associated with death avoidance. Experience of a deceased relative in family, death education, and family atmosphere of discussing death are positively associated with natural acceptance. Only religious belief is an influential factor and positively associated with approach acceptance. Gender and death education are negative, and religious belief is positive influential factors of escape acceptance.

### 3.4. Main Findings of the Present Study

Among the five domains of attitude towards death of the studied nursing interns, the average item score of the domain natural acceptance is the highest (3.67 ± 0.52), which is above 3 points and suggests that most of the nursing interns regarded death as a natural phenomenon in the life. They could accept death neutrally, neither welcome nor reject. The average item score of approach acceptance is the lowest (2.62 ± 0.52), which suggests that most of the nursing interns did not agree with the view that there is still life after death. In general, the nursing interns appeared to have a positive attitude towards death in general. Although their scores of natural acceptance and escape acceptance were statistically significantly lower the corresponding norms, their scores of death avoidance, fear of death and approach acceptance were statistically significantly higher (Table 1). The results suggest that the nursing interns might not regard death positively, and if they faced death, they might feel fear, avoidance and escapement. Therefore, the nursing educators should give the nursing interns more guidance and education on how to face unexpected death before they start internship.

## 4. Discussion

Previous study conducted in China indicated that factors associated with inpatient department nurses' attitude towards the care of dying patients include education level, fear of death, approach acceptance, religious beliefs, previous education on death and dying, natural acceptance, professional title, and experience with death or dying patients [19]. In our study, we mainly focused on following factors that might be associated with the nursing interns' attitude towards death.

### 4.1. Gender

This KAP survey showed that the scores of the domain escape acceptance of the male interns were higher than those of females. The possible reason might be that males have more psychological pressure on their future [22]. It suggests that more attention should be paid to the attitude towards death of male nursing students during teaching [12]. However, we should notice the small proportion (5.6%) of the males in current survey. It maybe was not a representative sample of male nursing students. The finding needs to be verified using a study with a larger sample size.

### 4.2. Religious Belief

In our survey, the nursing interns who had no religious belief showed higher scores in domains fear of death, approach acceptance, and escape acceptance, which was inconsistent with previous researches [23, 24]. However, after adjusting for other covariates and norm, the multiple regression analyses indicated that religious belief was a positive influential factor of fear of death, approach acceptance, and escape acceptance, which is consistent with previous researches that individuals with stronger religious belief showed less fear of death [19, 25]. Religious belief may serve as a comfort when facing death and help some nurse regard death as a normal process of life. Therefore, these nurses are calm rather than feared when facing death [23, 24]. However, we should realize that there is no unified religious belief in China; therefore, the cognition of death based on religion in China might be quite different from those in western countries. Furthermore, only 9.0% of the surveyed interns in the study had religious belief, which might bias the finding.

In summary, religious belief may have impact on nursing interns' attitude towards death, which suggests that nursing educators should try to know the death culture in different religious beliefs and nursing students' religious background before their nursing practices.

### 4.3. Family Atmosphere of Discussing Death

Studies showed that family atmosphere of discussing death played an important role in developing attitude towards death [18, 26]. Our study showed that the more openly and freely a family discussed about death, the more naturally the nursing interns accepted and faced death. The positive attitude towards death might be related to the environment where people grew up. Discussing death with family members could reduce fear, remove mysteriousness of death, and even an easy discussion itself may comfort people. Therefore, a free and open atmosphere of discussion about death in family could help to develop a natural and positive attitude of death. This also suggests that during the education for nursing interns, the educators should encourage the students actively joining the discussion about death-related issues, and expressing their views openly, listening to others carefully, and accepting different opinions. These will contribute to the development of a positive attitude towards death.

### 4.4. Experience of a Deceased Relative in Family

Few studies investigated the influence of the death of a relative on the attitude towards death. Our study showed that the nursing interns who experienced death of a relative in family could face death more calmly. It suggests that we could adopt scenario simulation, hospice ward visiting, or other ways in death education to let nursing students have more experience of death, and increase their understanding of dying patients, eliminate their fear, and make them calmer when facing dying patients.

### 4.5. Death Education

Some studies showed that the death education had positive impact on the attitude towards death, such as more calm and less fear [26, 27]. Death education maybe help nursing interns form a positive attitude towards death and reduce anxiety [28, 29]. But one research showed that there was no significant correlation between training courses about death and the fear of death [8]. This study showed that the scores of the domain natural acceptance of the nursing interns who had death education were significantly higher than that those who had no death education. In the domains fear of death, death avoidance, and escape acceptance, the scores of the nursing interns who had death education were significantly lower than those who did not.

If nursing interns who had no death education before and been exposed to dying patients in clinical practice, they would have difficulty to cope with the situation psychologically. Therefore, they might have more negative and passive reactions to death such as fear and avoidance. In our survey, less than half (46.5%) of the nursing interns had death education. It indicates the deficiency of the death education in the nursing education in China.

Nursing students would benefit from an educational program focused on caring for terminally ill people and their families [30, 31]. The education should be individualized and culturally sensitive in order to positively influence the students' attitude, and promote their professional development [32]. A number of Chinese medical schools and colleges have begun to offer courses related to death education and caring for dying patients. Interestingly, these courses were given by different departments, including nursing, sociology, psychology, health education, philosophy, education, counseling, and religious study [33]. 

We recognize that there are limitations in our study. The study is crosssectional, therefore, no causal relationship can be confirmed in the study. Our sample is a convenient sample, which might not represent the nursing intern population in China, even in the studied city. The sample size is small, which means the power might be low in some subgroup analyses. We have planned to replicate and confirm our findings using a prospective design with a larger random sample in the future. Because of the tradition concept in China that nurse is a job for women, there were only few male nurses could be recruited in our study. The limited number might hinder the generalization of our findings in male nurses.

## 5. Conclusion

The DAP‑R scores of attitude towards death are at a moderate level in the surveyed Chinese nursing interns. The positive attitude to death and death education before clinical practice are helpful to nursing interns when they care for dying patients. There is a lack of death education for nursing students in China, which should be reinforced in the future.

## Figures and Tables

**Table 1:** Comparison of the attitude domains' scores (mean ± standard deviation) between the norms and the nursing interns (*n* = 357).

Domain	Number of items	Domain score	Average item score	Rank	Norms [17]	*t*	*p* -value
Natural acceptance	5	18.36 ± 2.62	3.67 ± 0.52	1	21.95 ± 2.90	−25.91	0.001
Death avoidance	5	14.94 ± 3.30	2.99 ± 0.66	2	11.10 ± 4.80	22.00	0.001
Fear of death	7	20.34 ± 3.98	2.90 ± 0.57	3	19.04 ± 6.58	6.204	0.001
Escape acceptance	10	13.64 ± 3.64	2.73 ± 0.73	4	15.05 ± 4.75	−7.31	0.001
Approach acceptance	5	26.18 ± 5.16	2.62 ± 0.52	5	24.20 ± 7.80	7.24	0.001

**Table 2:** Comparison of the attitude domains' scores (mean ± standard deviation) between the subgroups by demographic characteristics.

Demographic group		n	Fear of death	Death avoidance	Natural acceptance	Approach acceptance	Escape acceptance
Gender	Male	20	21.20 ± 3.97	15.75 ± 2.82	18.25 ± 3.43	26.30 ± 7.57	15.45 ± .524
	Female	337	20.29 ± 3.94	14.89 ± 3.32	18.36 ± 2.57	26.10 ± 5.00	13.53 ± 3.56
Statistic			*t* = 0.991	*t* = 1.129	*t* = −0.190	*t* = −0.107	*t* = 2.30
*p*-value			0.322	0.260	0.849	0.916	0.022

Age	≤20	124	20.69 ± 4.12	15.18 ± 3.19	17.88 ± 2.65	25.6 ± 4.72	13.72 ± 3.51
	21–22	186	20.13 ± 3.99	14.6 ± 3.29	18.64 ± 2.59	26.20 ± 5.12	13.51 ± 3.70
	≥23	47	20.28 ± 3.50	15.30 ± 3.59	18.51 ± 2.51	27.3 ± 6.22	13.98 ± .380
Statistic			*F* = 0.758	*F* = 1.118	*F* = 3.271	*F* = 1.854	*F* = 0.358
*p*-value			0.469	0.328	0.039	0.158	0.700

Religious belief	Yes	32	20.14 ± 3.93	14.84 ± 3.23	18.29 ± .2.59	25.70 ± 4.76	13.41 ± .53
	No	325	22.44 ± 3.91	16.00 ± 3.83	19.06 ± .2.89	30.97 ± 6.52	16.03 ± .97
Statistic			*t* = −3.163	*t* = −1.911	*t* = −1.597	*t* = −0.750	*t* = −3.972
*p*-value			0.020	0.057	0.110	0.001	0.001

The only child in the family	No	245	20.42 ± 3.72	14.93 ± 3.30	18.16 ± 2.53	26.27 ± 4.76	13.7 ± 3.43
	Yes	112	20.18 ± 4.50	14.96 ± 3.31	18.80 ± 2.77	25.96 ± 5.96	13.5 ± 4.07
Statistic			*Z* = −0.527	*t* = −0.089	*t* = −2.182	*t* = 0.525	*t* = 0.496
*p*-value			0.598	0.96	0.03	0.60	0.60

Living with single parent	No	327	20.38 ± 4.03	15.02 ± 3.30	18.28 ± 2.59	26.14 ± 5.056	13.67 ± 3.57
	Yes	30	19.97 ± 3.39	14.07 ± 3.17	19.27 ± 2.80	26.57 ± 6.27	13.30 ± 4.40
Statistic			*t* = 0.544	*t* = 1.520	*t* = 0.565	*t* = −0.432	*t* = 0.536
*p*-value			0.587	0.129	0.407	0.67	0.592

Living area	City	99	20.85 ± 3.88	15.29 ± 2.80	18.39 ± 2.50	26.14 ± 4.48	12.95 ± 3.53
	Town	141	20.39 ± 4.29	15.05 ± 3.32	18.54 ± 2.85	26.01±5.93	14.06 ± 3.95
	Countryside	117	19.86 ± 3.62	14.513.63	18.11 ± 2.43	26.40 ± 4.71	13.73 ± 3.27
Statistic			*F* = 1.671	*F* = 1.632	*F* = .865	*F* = 0.183	*F* = 2.765
*p*-value			0.190	0.197	0.422	0.833	0.064

Education level	Undergraduate	200	20.25 ± 3.98	14.72 ± 3.21	18.60 ± 2.62	26.21 ± 5.52	13.50 ± 3.65
	Graduate	55	20.09 ± 3.75	15.56 ± 3.68	18.33 ± 2.53	26.60 ± 4.61	13.64 ± 3.54
	Other	102	20.67 ± 4.10	15.05 ± 3.23	17.902.63	25.88 ± 4.72	13.93 ± 3.69
Statistic			*F* = 0.503	*F* = 1.509	*F* = 2.423	*F* = 0.354	*F* = 0.484
*p*-value			0.605	0.223	0.090	0.702	0.617

Perception of mind and body	Poor	40	19.38 ± 3.66	14.05 ± 2.98	18.98 ± 2.61	25.65 ± 4.13	14.53 ± 4.06
	Moderate	178	20.60 ± 3.76	14.80 ± 3.18	17.89 ± 2.49	26.28 ± 4.79	13.79 ± 3.22
	Good	122	20.30 ± 4.42	15.34 ± 3.57	18.82 ± 2.65	26.22 ± 5.83	13.21 ± 4.15
	Very good	17	20.24 ± 3.46	15.71 ± 2.78	18.53 ± 3.06	26.06 ± 6.29	13.12 ± 2.50
Statistic			*F* = 1.051	*F* = 1.990	*F* = 4.042	*F* = 0.165	*F* = 1.567
*p*-value			0.370	0.115	0.008	0.920	0.197

Experience in caring for dying patients	No	103	20.98 ± 3.62	15.20 ± 3.17	17.83 ± 2.60	26.41 ± 5.47	14.00 ± 3.63
	Yes	254	20.09 ± 4.09	14.83 ± 3.35	18.57 ± 2.60	26.08 ± 5.04	13.50 ± 3.64
Statistics			*t* = 1.934	*t* = 0.958	*t* = −2.467	*t* = 0.539	*t* = 1.186
*p*-value			0.054	0.339	0.014	0.590	0.237

Experience of a deceased relative in family	No	123	20.33 ± 3.78	14.79 ± 3.10	17.80 ± 2.53	25.79 ± 4.67	13.13 ± 3.49
	Yes	234	20.35 ± 4.08	15.02 ± 3.40	18.65 ± 2.63	26.38 ± 5.40	13.91 ± .70
Statistics			*t* = −0.039	*t* = −0.633	*t* = −2.970	*t* = −1.030	*t* = −1.932
*p*-value			0.969	0.527	0.003	0.304	0.054

Death education	No	191	21.10 ± 4.09	15.61 ± 3.12	17.81 ± 2.43	26.33 ± *4.55*	14.01 ± .31
	Yes	166	19.48 ± 3.65	14.17 ± 3.334	18.99 ± .69	26.00 ± *5.79*	13.22 ± 3.95
Statistic			*t* = 3.929	*t* = 4.188	*t* = −4.381	*t* = 0.602	*Z* = −2.503
*p*-value			0.001	0.001	0.001	0.548	0.012

Family atmosphere of discussing death	Open and free	130	19.15 ± 4.51	13.80 ± 3.64	19.79 ± 2.41	25.84 ± 6.01	13.43 ± 4.47
	Awkward, uncomfortable	35	21.86 ± 2.88	15.51 ± 2.58	17.29 ± 2.24	25.86 ± 4.44	13.14 ± 3.02
	Avoiding	71	21.83 ± 3.31	16.01 ± 2.84	18.21 ± 2.55	26.77 ± 4.90	14.18 ± 3.06
	Only when necessary, and avoiding children and elderly	121	20.31 ± 3.57	15.37 ± 3.01	17.21 ± 2.24	26.28 ± 4.47	13.69 ± 3.07
Statistic			*χ* ^2^ = 7.212	*χ* ^2^ = 18.27	*χ* ^2^ = 48.70	*F* = 0.563	*F* = 0.896
*p*-value			0.065	0.001	0.001	*0.639*	0.440

**Table 3:** Generalized linear regression analysis of the nursing interns' attitudes towards death.

Domain	Independent variables	adjusted *β*	*β*	*t*	*p*-value
Fear of death	Norm	2.988		74.087	0.001
	Religious belief	0.355	0.179	3.487	0.001
	Death education	−0.244	−0.215	−4.196	0.001

Death avoidance	Norm	3.513		32.422	0.001
	Death education	−0.249	−0.189	−3.681	0.001
	Family atmosphere of discussing death	−0.138	−0.204	−3.986	0.001

Natural acceptance	Norm	3.541		24.104	0.001
	Experience on a deceased relative in family	0.144	0.131	2.762	0.006
	Death education	0.182	0.173	3.628	0.001
	Family atmosphere of discussing death	0.147	0.362	7.601	0.001

Approach acceptance	Norm	2.570		93.787	0.001
	Religious belief	0.526	0.292	5.750	0.001

Escape acceptance	Norm	3.086		18.719	0.001
	Gender	−0.339	−0.107	−2.076	0.039
	Religious belief	0.519	0.204	3.943	0.001
	Death education	−0.181	−0.124	−2.413	0.016

## Data Availability

The data used to support the findings of this study are available from the corresponding author upon request.
